# A comparative analysis of experienced uncertainties in relation to risk communication during COVID19: a four-country study

**DOI:** 10.1186/s12992-022-00857-x

**Published:** 2022-06-27

**Authors:** Florin Cristea, Heide Weishaar, Brogan Geurts, Alexandre Delamou, Melisa Mei Jin Tan, Helena Legido-Quigley, Kafayat Aminu, Almudena Mari-Sáez, Carlos Rocha, Bienvenu Camara, Lansana Barry, Paul Thea, Johannes Boucsein, Thurid Bahr, Sameh Al-Awlaqi, Francisco Pozo-Martin, Evgeniya Boklage, Ayodele Samuel Jegede, Charbel El Bcheraoui

**Affiliations:** 1 Evidence-Based Public Health Unit, Centre for International Health Protection, Robert Koch Institute, Nordufer 20, 13353 Berlin, Germany; 2 African Center of Excellence for the Prevention and Control of Communicable Diseases & Centre de Formation et de Recherche en Santé Rurale de Maferinyah, PoBox 1017, Dixinn, Conakry, Guinea; 3 Saw Swee Hock School of Public Health, National University of Singapore, 12 Science Drive 2, #10-01, Singapore, 117549 Singapore; 4 Department of Sociology, Faculty of the Social Sciences, University of Ibadan, 1, Oyo Road, Agbowo, Ibadan, Nigeria; 5 Centre for International Health Protection, Robert Koch Institute, Nordufer 20, 13353 Berlin, Germany; 6 Centre de Formation et de Recherche en Santé Rurale de Maferinyah, Département de Recherche, Unité de Socio-Anthropologie, Conakry, Guinea; 7 African Center of Excellence for the Prevention and Control of Communicable Diseases, PoBox 1017, Dixinn, Conakry, Guinea; 8 Department of Infectious Disease Epidemiology, Robert Koch Institute, Nordufer 20, 13353 Berlin, Germany; 9 Postgraduate Training for Applied Epidemiology (PAE), Robert Koch Institute, Berlin, Germany; 10 European Programme for Intervention Epidemiology Training (EPIET), European Centre for Disease Prevention and Control (ECDC), Stockholm, Sweden; 11 Health Information Centre for International Health Protection unit, Centre for International Health Protection, Robert Koch Institute, Nordufer 20, 13353 Berlin, Germany

**Keywords:** Risk communication and community engagement, Uncertainty, Risk assessment, Trust

## Abstract

**Background:**

During outbreaks, uncertainties experienced by affected communities can influence their compliance to government guidance on public health. Communicators and authorities are, hence, encouraged to acknowledge and address such uncertainties. However, in the midst of public health crises, it can become difficult to define and identify uncertainties that are most relevant to address. We analyzed data on COVID-19-related uncertainties from four socio-economic contexts to explore how uncertainties can influence people’s perception of, and response to Risk Communication and Community Engagement (RCCE) strategies.

**Results:**

This qualitative study, which adopts an interpretative approach, is based on data from a documentary review, key informant interviews (KII), and focus group discussions (FGD) with members of the general public and people with barriers to information from Germany, Guinea, Nigeria, and Singapore. Transcripts from the KII and FGD were coded and analyzed thematically. We interviewed a total of 155 KIs and conducted 73 FGD. Our analysis uncovered a divergence between uncertainties deemed relevant by stakeholders involved in policy making and uncertainties that people reportedly had to navigate in their everyday lives and which they considered relevant during the pandemic. We identified four types of uncertainties that seemed to have influenced people’s assessment of the disease risk and their trust in the pandemic control strategies including RCCE efforts: epidemiological uncertainties (related to the nature and severity of the virus), information uncertainties (related to access to reliable information), social uncertainties (related to social behavior in times of heightened risk), and economic uncertainties (related to financial insecurities).

**Conclusion:**

We suggest that in future outbreaks, communicators and policy makers could improve the way in which affected communities assess their risk, and increase the trust of these communities in response efforts by addressing non-epidemiological uncertainties in RCCE strategies.

## Background

When the World Health Organization (WHO) declared the COVID-19 outbreak a Public Health Emergency of International Concern on the 30th of January 2020 and later a pandemic on the 11th of March 2020, countries were only beginning to understand the characteristics and the behavior of the virus. Nevertheless, the lack of available evidence and the rapid evolution of the knowledge related to COVID-19 and its spread created significant challenges to risk communication globally since the beginning of January 2020 [1], as initial information from Wuhan started to become of interest. Months of uncertainties ensued and persisted until today. Much more is now known about the virus’s nature which has allowed countries to adjust their measures to cope with the pandemic. However, the consequences of the uncertainties have brought about challenges to populations globally. Recent studies show that COVID-19 related uncertainties can contribute to negative psychological effects, such as anxiety and stress, with potential long-lasting effects on those who experience these symptoms [2, 3]. Particularly, stress experienced during the pandemic is associated with less compliance [4]. Likewise, in previous infectious disease outbreaks, such as Ebola, Zika, and the H1N1 virus, experienced uncertainties and barriers to information have contributed to reduced public adherence to preventive measures [5–9]. Appropriate communication about the knowns and unknowns of an outbreak, therefore, becomes of utmost importance to avoid confusion with, and reluctance to, recommended public health measures among affected communities.

Uncertainty remains a constant characteristic of the COVID-19 pandemic [10] and requires effective communication to manage the evolving dynamics of the virus. However, the relationship between experienced uncertainties and risk communication efforts was complicated by the surge of misinformation during the outbreak [11]. Previous research showed that the proliferation of misinformation, which often bears contradictory messages and is disseminated through multiple sources, can weaken the state of certainty [12]. Moreover, the contradictory discourses can increase uncertainty and delay policy actions as efforts are redirected to verify the information [13]. When uncertainty is not addressed in a timely manner, it can result in rumors in the public domain [1, 14] and may erode Risk Communication and Community Engagement (RCCE) efforts. In order to better manage infectious disease outbreaks, understanding the relationship between the perceived uncertainties and the information people acquire is crucial [15].

Numerous scholars have attempted to advance a working definition of uncertainty that could be operationalized across disciplines [16, 17]. We understand uncertainty as “a dynamic state in which there is a perception of being unable to assign probabilities for outcomes that prompts a dis-comforting, uneasy sensation that may be affected (reduced or escalated) through cognitive, emotive, or behavioral reactions, or simply by the passage of time and changes in the perception of circumstances.” [18] To disentangle the attributes of the concept of uncertainty from the characteristics of the lived experience of uncertainty [18], we follow Abdellaoui and colleagues [19] distinction between described uncertainty and experienced uncertainty. The first refers to contexts where alternatives for decision making are described. The latter to contexts where the decision maker’s knowledge of possible outcomes is incomplete. This focus on experienced uncertainty sets the framework we propose apart from other types of uncertainty discussed in the literature. Previous examples include epistemic uncertainty (about facts, numbers, and science) [20] or aleatory uncertainty (inevitable unpredictability of the future due to unforeseeable factors, commonly used in statistical modeling of risk) [21].

The literature often draws attention to the link between experienced uncertainty and negative health outcomes caused by stress and anxiety. Adverse psychological effects caused by uncertainty have been described concerning invisible contaminants [22]. In the context of illness and hospitalization, studies showed that uncertainty about symptoms and outcomes serves as a predictor for increased stress (prominently [23]). Data from the H1N1 pandemic links high uncertainty-intolerance to increased anxiety and stress [24]. And one study associates high levels of hope with low levels of experienced uncertainties among survivors of breast cancer [25].

In this pre-COVID uncertainty literature, one focus is on how to best communicate uncertainty to the public. It is suggested that communicating uncertainty does not necessarily impact audiences negatively, but readers are cautioned that the impact of this communication varies between individuals and communication formats, as well as the emergency situation itself [20, 26]. For instance, adjusting communication practices to information seeking behaviors that emerge in a state of uncertainty was described as one efficient means of managing uncertainty during the HIV epidemic [27]. Additionally, reflections from previous pandemics showed that inconsistent and ineffective information concerning scientific uncertainty in narratives from the WHO and news coverage can increase the anxiety of affected populations, further highlighting the gaps in our knowledge about communicating uncertainty [28–30].

Since the start of the COVID-19 pandemic, we have observed an amplified interest in the study of uncertainty, with most studies reporting an increase of anxiety and stress, in the community, caused by experienced uncertainty [3, 31]. Some of these studies showed that addressing uncertainty about COVID-19 can be linked with improved health-outcomes among cancer patients [32], while others showed that lower tolerance to uncertainty was associated with lower intentions to get vaccinated [33]. Experimental studies conducted during the pandemic suggest that addressing outcome uncertainty and scientific uncertainty may not persuade people to get vaccinated [34] and may not produce behavioral responses [35]. Therefore, additional research on uncertainty message framing is needed that is sensitive to the impacts of situational factors, such as the socio-cultural context, level of education, and income of populations targeted in communications [34]. Furthermore, notwithstanding the growing research interest in uncertainty, we agree with Afifi and Afifi [36] that despite the health- and decision-related implications following an increase of experienced uncertainty, there is very little empirical investigation around the experience of uncertainty during the COVID-19 pandemic, when compared to the actual need of such studies.

In this paper, we explore what kind of uncertainties the current pandemic generated and how these uncertainties influenced public perception of, and response to, RCCE in Germany, Guinea, Nigeria, and Singapore. Our study also seeks to understand the extent to which these were addressed by relevant authorities during the outbreak, and if and how addressing these uncertainties could improve RCCE.

## Methods

We employed qualitative research methods, adopting an interpretative approach for data analysis. We collected data with three interconnected methods: document review, key informant interviews (KII), and focus group discussions (FGD). The study focused on four countries: Germany, Nigeria, Guinea and Singapore. The selection of countries was based on previous collaborations between the partner institutions, on early technical exchange and on the advantage presented by the broad variety in sociocultural, political, and epidemiological contexts from the participating countries with different experiences responding to public health emergencies and outbreaks. At the time of fieldwork, the four countries were in the midst of different pandemic phases. Germany was heading towards or experiencing a second lockdown. As a consequence of low infection rates, both Singapore and Guinea were at the stage of easing restrictions. Likewise, Nigeria was easing restrictions although the national government was warning against a second wave.

Building up to these different pandemic phases, each country underwent varying emergency levels concerning the exponential growth of COVID-19 cases. Germany went through an initial wave (March – May 2020) that predominantly affected young and middle-aged adults. These cases were reportedly mild compared to the impact of an infection in people over the age of 60. However, especially in patients over 80 years old, every second case resulted in hospitalization, and one in three ended in death [37]. If during the first wave, the incidence level barely exceeded 5000 cases a day, during the second wave (October 2020 – Mar 2021) [38], the incidence peaked at over 30.000 a day in December [39], while we were still conducting interviews and FGDs. Nigeria also went through two epidemic waves (February – October 2020; November 2020 – February 2021) [40]. Nevertheless, there were significantly less reported cases (barely reaching 600 cases a day during the first wave), with a very low case fatality rate [41]. Singapore confronted up to the point of data collection a single wave (approximately April 2020), with several hundred cases a day, barely exceeding 1000 cases at its peak, with a very low fatality rate, and migrant workers in dormitories being most affected by the outbreak [41]. Finally, Guinea also experienced a single wave (April – October 2020), not exceeding 300 cases a day at its highest and a very low death rate caused by an infection [42].

### Documentary review

Each country team conducted a content analysis of documentary data to gain an understanding of the national and regional RCCE responses to COVID-19 and to identify key stakeholders involved in RCCE. Documents and relevant material were collected through online searches of key websites and other sources, such as the websites of national and regional Ministries of Health and institutes of public health, other relevant ministries and agencies, international and non-governmental organizations, and COVID-19-specific campaigns and strategies. We followed a content analysis framework using a set of pre-defined categories derived from the existing RCCE literature to analyze the retrieved documents.

### Primary data collection

Each country team conducted semi-structured KII with stakeholders involved in the design and implementation of RCCE at sub-national and national levels. In addition, FGD were held with members of the general public and with groups of individuals that experienced barriers to understanding COVID-19 related messages or engaging in prevention measures. Interview guides were developed based on a review of RCCE theories and existing literature, results of the document review, and several initial scoping interviews. Amendments were made to the interview guides during data collection in order to suit the local context, the evolving pandemic situation, and the individual interviewees and focus group discussants. Due to the COVID-19 situation and related contact and travel restrictions, KII and FGD were conducted either in-person (with required distance, masks, and ventilation) or online. All KII and FGD were recorded, transcribed verbatim, and translated into English if conducted in another language. All participants were provided a consent form to sign as agreement to participate. Some KIs from Nigeria only provided verbal consent.

The interview guide for KII covered four categories: involvement and role in RCCE efforts (e.g. reasons for participation in the risk communication response), strategy and design of RCCE (e.g. how messages were developed), public and community engagement (e.g. perception of community response), and sustainability (e.g. incorporation of feedback in the continuous RCCE strategy). Each country team initially selected key informants through purposive sampling, with additional participants being included through snowball sampling. In total, 155 individuals were interviewed during 142 recorded KII. Some interviews included more than one individual. Interviews lasted on average 53 minutes.

The topic guide for FGD covered five categories: knowledge and understanding of COVID-19 messages (e.g. what were the perceived main messages), assessment of risk (e.g. rating dangerousness of an infection), information sources and engagement initiatives (e.g. community responses to the outbreak), sociocultural factors influencing understanding of messages (e.g. language barriers), and public engagement (e.g. responses to health recommendations). Each team recruited focus group participants using convenience sampling. Two distinct groups were selected: (1) the general public and (2) groups that experienced barriers to understanding COVID-19 related messages or to engaging in prevention measures. Representatives of the general public included people over the age of 60 (all four countries), parents of young children (in Germany), young adults (18–30 years old) (in Germany, Guinea, and Singapore), community health workers (in Guinea), and people in different employment situations (in Nigeria and Singapore). The FGD with people who were identified to have barriers to information focused on those with a migration background and limited language proficiency of national languages (in Germany and Singapore), people with low education status (in Guinea), and regional ethnic minorities (in Nigeria). The participants we selected reflect typologies described in the literature to suffer from structural and socio-cultural barriers to relevant information [43, 44]. Furthermore, we included this purposive sample of FGD participants based on each team’s knowledge of local contexts and the documentary review and tried to identify country-specific members of the population that appeared to have been affected more by the pandemic. Seventy-three FGD were conducted with 419 individuals. FGD lasted on average 63 minutes. Researchers conducted FGD in the national, local, or migrants’ native languages, and were supported when necessary by a translator.

Due to pandemic restrictions, in Singapore, researchers conducted 16 one-to-one individual interviews using a modified topic guide with members of the general public and people experiencing barriers to understanding COVID-19 related messages or engaging in prevention measures.

### Data analysis

Data were analyzed using inductive and deductive thematic content analysis in NVIVO R1 (QSR International, 2020). A coding scheme was developed based on the KII and FGD topic guides, pre-existing RCCE literature, and a team discussion following co-coding of a subset of the data which helped to align codes. The jointly agreed coding scheme was then systematically applied to the entire data set.

Once RCCE relevant topics were identified from the initial analysis and authors were familiarized with the data, we engaged in a second line of analysis to determine the relationship between RCCE efforts and uncertainties generated by the pandemic. During public health emergency events, it is difficult to discern relevant from irrelevant uncertainties, and the literature provides no consensus about what uncertainties should be of primary concern for policy makers and health authorities [13]. To circumvent this difficulty, we used techniques from the grounded theory approach to determine the framework for our analysis [45]. For the entirety of this round of analysis, we organized regular meetings, during which we discussed emerging codes, resolved disagreements, and compared codes to reach consistency. We considered the full transcript segment mentioning uncertainty to be a unit of analysis. This second line of inquiry included three steps: First, individual coders looked for and summarized segments from the transcripts where uncertainties related to the pandemic experienced by affected communities were described. The summaries of the segments included the main causes for the uncertainties (e.g. duration of the pandemic, economic concerns, being unsure where to find relevant information etc.). Second, we developed categories of uncertainties by comparing and organizing the initial summaries, accounting for content and frequency. For example, if several summaries included economic concerns as the main cause for the uncertainties described, they were grouped into a single category. Third, after comparing and establishing the relationships between the categories, we organized these into the following four types that informed the framework for our analysis: epidemiological uncertainties, information uncertainties, social uncertainties, and economic uncertainties. We could not identify other types of uncertainties.

Epidemiological uncertainties relate to the nature or the spread of the virus. Information uncertainties refer to an inability to identify reliable sources of information or not knowing where to access necessary information. Social uncertainties refer to those generated within an individual’s immediate social environment with regards to an inability to predict one’s own behaviors in relation to other people’s behavior (see also [46, 47]). Economic uncertainties are those associated with financial insecurities either of individuals, their families, or the whole country. Once we established the framework for our analysis, we compared how key informants and focus group participants who reported uncertainties responded to topics identified in the first line of analysis. These included, but were not limited to, questions about the risks of the disease, awareness and understanding of messages, emotional responses to messages, adherence and reluctance to containment rules and recommendations. We considered theoretical saturation once the comparison between the uncertainty framework and the initial coding scheme did not reveal any new themes. The two main themes that emerged out of our analysis were: first, divergence between the uncertainties deemed relevant by key stakeholders involved in RCCE and the uncertainties reported by members of the general public and persons with barriers to information; second, the relationship between unaddressed uncertainties and the individual ability to assess risk on one hand, and the trust placed in the overall pandemic response on the other hand.

## Results

The fieldwork was undertaken between August and December 2020. We conducted 56 KII in Germany, 38 in Guinea, 46 in Nigeria, and 15 in Singapore. KII are detailed in Table 1. We conducted 26 FGD in Germany, 22 in Guinea, 12 in Nigeria, and 13 in Singapore. In addition, we conducted 16 individual interviews with members of the general public and persons experiencing barriers to information in Singapore. FGD and individual interview participants are detailed in Table 2.

**Table 1:** Summary of key informant interviews

Types of key informants	Germany (*n* = 56)	Guinea (*n* = 38)	Nigeria (*n* = 46)	Singapore (*n* = 15)	Total (*n* = 155)
*Academic*	*6*	*1*		*7*	*9% (14)*
*Community representative*	*1*	*6*	*12*		*12% (19)*
*Health care professional*	*1*	*3*	*1*		*3% (5)*
*International / Intergovernmental organization representative*		*6*	*9*		*10% (15)*
*Media representative*	*4*	*5*	*2*		*7% (11)*
*Non-governmental organization representative*	*11*	*6*	*6*	*5*	*18% (28)*
*Political decision maker*	*13*	*1*			*9% (14)*
*Public health administration / authority representative*	*20*	*10*	*16*	*3*	*32% (49)*

**Table 2:** Summary of focus groups and individual interviews

	Germany (*n* = 26)	Guinea (*n* = 22)	Nigeria (*n* = 12)	Singapore (*n* = 13)	Individuals in Singapore	Total (*n* = 73)
*Participant groups*						
*General Public*	*20 (n = 112)*	*11 (n = 67)*	*7 (n = 61)*	*10 (n = 32)*	*n = 12*	*48 (n = 284)*
*Groups with barriers to information or participation*	*6 (n = 34)*	*11 (n = 66)*	*5 (n = 36)*	*3 (n = 11)*	*n = 4*	*25 (n = 151)*
*Median Age (range)*						
*General Public*	*47 (18–80)*	*33 (20–75)*	*37 (22–60)*	*30 (21–65)*	*30 (23–56)*	*35.40 (18–80)*
*Groups with barriers to information or participation*	*45 (24–84)*	*36 (18–85)*	*33 (18–51)*	*not provided*	*not provided*	*38 (18–85)*
*Gender*						
*Male*	*62*	*75*	*49*	*19*	*10*	*215*
*Female*	*83*	*58*	*48*	*24*	*6*	*219*
*No answer*	*1*					*1*

Uncertainties were a major problem reported by our study participants. In the following sections, we will describe first how the uncertainties addressed by regional and national health authorities in their public communications diverged from those experienced by the general public and vulnerable groups. Second, we will detail the types of uncertainties we found and their connection to people’s reception of RCCE efforts. Specifically, we portray how these uncertainties, if unaddressed, were often negatively associated with people’s reported ability to assess the health risk posed by the SARS-CoV-2 virus and their trust in containment efforts of regional and national authorities.

### Gap between uncertainties acknowledged by authorities and those experienced by affected communities

Most stakeholders involved in RCCE and mitigation efforts in all four countries acknowledged heightened uncertainty in the population. Key informants from Germany, Nigeria, and Singapore attempted to address these uncertainties during public communications as part of their RCCE strategy. One interviewee from Nigeria, summed up these efforts:

*“In risk communication, part of what you communicate is uncertainty. So, you are telling people what to do and then you're telling them you don't know much about it. ‘This is what we know, it could change.’ Even from the communication we were putting out, it was always, ‘as [we] know more, we will tell you more.’ This is a novel disease. Scientists were still [learning] about it. [ … ] Even us the communicators, we knew we didn't know everything about the disease.”*

People involved in the RCCE strategy in Germany and Nigeria, as well as scientists from Germany and Singapore identified as relevant communicators during the pandemic, considered the admission of epidemiological uncertainty an important strategic objective to convey transparency. They viewed transparency to be important for an increase in trust, which they considered necessary to foster better understanding and enhanced compliance in the general public. However, our analysis determined that what were considered relevant uncertainties at the level of policy makers and scientists informing policies were not always consistent with the uncertainties people described they had to navigate in their everyday lives and which they considered relevant during the pandemic. We found that unaddressed and unresolved epidemiologic and non-epidemiological uncertainties influenced people’s ability to assess their health risk and their trust in containment and RCCE efforts. We identified the following uncertainties, divided by country and by type of uncertainties (Table 3):

**Table 3:** Sources of uncertainties by country and by type of uncertainties

	Germany	Guinea	Nigeria	Singapore
*Epidemiological uncertainties*	*Outcome of an infection. Comprehensibility of regulations to contain the spread.*	*Severity of an infection. Comprehensibility of regulations to contain the spread.*	*Severity of an infection.* *Comprehensibility of regulations to contain the spread.*	*Outcome of an infection.* *Accuracy of received information about the virus (asymptomatic carriers, modes of transmission, long Covid).*
*Information uncertainties*	*Navigating high amount of information.* *Identifying reliable communicators.* *Discrepancy between governmental recommendations and expert opinions.*	*Navigating high amount of information.* *Identifying reliable communicators.*	*Navigating high amount of information.* *Identifying reliable communicators.*	*Navigating high amount of information.*
*Social uncertainties*	*Safely engaging in community life.* *Maintaining control of individual’s life.*	*Safely engaging in community life.* *Maintaining control of individual’s life.*	*Safely engaging in community life.* *Maintaining control of individual’s life.*	*Safely engaging in community life.* *Maintaining control of individual’s life.*
*Economic uncertainties*	*Impact of the pandemic on the economic stability of the country.* *Ability to economically sustain oneself and dependants.* *Accessing economic support provided by local governments (in case of job loss, or work-inability)*	*Ability to economically sustain oneself and dependants.* *Accessing material support provided by governments during quarantine.* *Resilience of the national healthcare system.*	*Ability to economically sustain oneself and dependants.* *Accessing material support provided by governments during quarantine.*	*Impact of the pandemic on the economic stability of the country.* *Ability to economically sustain oneself and dependants.* *Accessing economic support provided by local governments (in case of job loss, or inability for employment)*

### Uncertainties, risk assessment, and trust

#### Epidemiological uncertainties

Key informants considered that addressing epidemiological uncertainties as an important part of RCCE strategies. Nevertheless, focus group participants often raised uncertainties related to the transmission of the virus, its severity, and the comprehensibility of regulations implemented to contain the spread. Across countries, epidemiological uncertainties seemed to influence people’s perceived ability to assess the risk of infection and disease progression. Furthermore, participants reporting epidemiological uncertainties differed in terms of the trust they placed in RCCE and governmental containment efforts across socio-economic contexts.

Most focus group participants from Singapore evaluated the risk associated with a COVID-19 infection as moderate. This was commonly reported across migrants with limited national language proficiency, as well as young adults participating in the study. They expressed trust in the Singaporean health system and received the risk communication efforts of local authorities positively. Nevertheless, a small number of participants still expressed uncertainties about the impact of the pandemic and the outcome of an infection. These participants reportedly felt uneasy regarding the information they had received about the virus, such as the impact of asymptomatic carriers, modes of transmission of the virus, and the long-term effects of an infection. Participants indicated that because of such uncertainties, they were insecure about how to behave in crowded areas or when meeting and/or having to care for friends and family members, and were unable to predict the consequences of the pandemic. Furthermore, participants who mentioned uncertainties regarding the virus and containment measures reported more often an increased risk perception compared with the overall group of research participants. One focus group participant from Singapore explained:

*“So, it’s hard for us to really find like the treatment for it. If vaccines and all - it seems like prevention strategies to lower, like, the cases [ … ] we’re all trying to contain, like, the rapid spread of [the virus]. So, until we find a perfect cure for it, I think it will always be scary because we don’t know the exact side effects.”*

Research participants from both Nigeria and Guinea found it difficult to assess the severity of the COVID-19 virus. Several participants dismissed the severity of an infection due to the low case fatality rate and the suspicion that political interests drove the pandemic response. Participants from both countries perceived that the imposition of lockdown and regulations was a disproportionate response to the actual threat caused by COVID-19. They compared the current response to previous disease outbreaks, where no lockdown had been imposed and where an infection had manifested with more severe symptoms. The examples given included outbreaks of the Lassa Fever, Ebola, and Malaria. One focus group participant from Guinea described the reason for his reluctance regarding the severity of the outbreak:

*“Yes! That's why I didn't believe in this disease! They say there are cases but they don't see any deaths! [They] say every day that there are sick people in Donka [A University Teaching Hospital in the capital city] but you don't see any death!”*

When participants from Nigeria and Guinea evaluated the governmental containment efforts, they did not refer to RCCE messages about the spread of the virus and how they can protect themselves (aimed at in RCCE communication). They assessed the overall response to contain the outbreak based on the impact the response had on people’s immediate social environment (e.g. loss of income, closing of churches and mosques, social distance requirements). Only in few cases they related the perceived danger posed by the virus on its contagiousness and the impact the outbreak had on other countries.

#### Information uncertainties

In all four countries, participants described having to deal with a high amount of information related to the SARS-CoV-2 virus. Particularly in Germany, two aspects contributed to uncertainties related to information described by focus group participants. First, participants explained that they were unsure about where to access reliable information about the virus and the regulations. Second, many participants from Germany struggled with navigating the diverse expert opinions that were not always aligned with official governmental recommendations. This was common not only across participants with migrant background or language barriers, but even more among native German speakers. One focus group participant from Germany explained:

*“I don't know at all which information that is issued today will still be valid tomorrow. [ … ] And to come back to the one example: I haven't received an answer to it until today. Do I only have to wear a mask if the minimum distance cannot be kept or do I always have to wear a mask? Some people say that if the minimum distance cannot be kept, you have to wear a mask, others say that the minimum distance doesn't matter at all, you always have to wear a mask here and there. I miss such truly clear things.”*

Having to deal with such uncertainties, combined with a perceived lack of available authoritative sources of information, and an inability to resolve their uncertainties through existing information channels, contributed to a situation where some participants were unsure about how to accurately assess the risk posed by the virus. Interestingly, these uncertainties related to information did not seem to influence the trust participants from Germany placed in the pandemic response. Similar to Singapore, they reported high trust when evaluating the authorities’ containment efforts.

Some particularities emerged out of key informant interviews with representatives of migrant organizations that described some uncertainties related to unequal access to information of people with a migration background in Singapore and Germany that were not apparent during FGDs. In Singapore, one key informant described how additional efforts were required to inform migrant workers because they were reluctant to get swabbed due to fears of losing their legal status in case of a positive test result. According to one key informant from Germany, people living in a refugee center that was closed based on few identified cases, experienced increased anxiety, an inability to accurately assess the risk, and an inability to know how they can protect themselves. The key informant explained that the ensuing uncertainties could have been avoided if more effort would have been invested to inform the affected community living in the respective center about what caused the closure of the refugee center and how long the measure was going to last.

*“And there was real anxiety amongst the refugees, who wondered what it meant for them, “what risks are we facing right now?”, “how can we protect our kids?”, there was no information for them. And then it seemed like the initial reception centers were closing even though there were still people in them, and there was no real effort to inform the refugees which led to massive anxiety amongst them about how they could protect themselves [ … ]”*

#### Social uncertainties

Participants from all four socio-economic contexts reported uncertainties about how to act and safely participate in social life during a time of heightened risk. Focus group participants from all four countries found it difficult to adjust to an uncertain social environment that was constantly changing due to the dynamic spread of the virus, and to ensuing regulations implemented to contain the spread. People felt that they were no longer in control of aspects of their own lives on which they felt they had some degree of autonomy before the pandemic. One focus group participant from Germany, briefly summed up their frustration:

*“You don't even plan anything anymore. In doubt you just leave it, because you do not know how anything will work.”*

Furthermore, the fear of asymptomatic carriers and uncertainties about how to follow protective measures in social encounters contributed to insecurities about how to protect oneself and others during social interactions. As a consequence, some participants reported an increased sense of fear. One member from the general public from Singapore, when asked about how he felt about the messages published by relevant authorities, stated:

*“Yeah, it also made me feel a bit fearful because it's asymptomatic, right? So, you don't know who you met along the street or even with friends and gatherings, you don't know who might actually have it. And even you, yourself, you're like, hey, today, maybe I actually have it.”*

Furthermore, the resulting insecurity about the probability to get infected in one’s immediate social environment was perceived as a threat to one’s ability to plan for every-day necessities, such as going to the market or continuing working in spaces where the recommended distance could be upheld. This, in turn, intensified already existing insecurities about the economic stability of affected families and communities. A related particularity emerged in Guinea and Nigeria, where several participants reported that doubt about how to safely maintain social interactions contributed to a perceived increase of distrust within local communities. One focus group participant from Guinea described the emerging mistrust within their working environment:

*“At that time, I had enough apprentices in my workshop, but now they came in rotation. If three come today and two tomorrow, and so on, that's how we did it until even mistrust set in between us. [ … ] No one was working [anymore], we were sitting at home.”*

#### Economic uncertainties

Participants across countries raised concerns about their ability to care for themselves or their families, and how to cope with economic uncertainties. More substantially, members of the general public often associated related uncertainties with an unease about the duration of the pandemic and its potential negative impact on the country’s economy, their job safety, or opportunities for future employment. Furthermore, participants from all four countries described uncertainties related to accessing funds (e.g. financial support for people losing their jobs in Singapore and Germany) and material support (e.g. food supplies in Nigeria and Guinea) provided by the government during the pandemic. Some participants from Guinea further expressed concerns about the stability of their healthcare system. While economic concerns were prevalent across countries, they seemed to be particularly high among participants from Nigeria and Guinea, who perceived the experienced uncertainties as an immediate existential threat. A Nigerian focus group participant explained:

*“COVID-19 has affected our daily lives most especially, the breakdown we’ve gotten, this kind of breakdown that everything was ceased, they sent us home, everybody went back home and nothing like business was going on, everybody was very poor, there’s no money for you to realize [something], even to eat, it was the biggest problem. Even the money you [need] to eat has been problem so how can you talk about making other arrangements of your life?”*

Participants from all four countries considered it was the responsibility of the state to make sure the basic needs of the population were met, if authorities expected the people to comply with regulations. In Germany, Guinea, and Nigeria, several participants related their unaddressed economic uncertainties to questions of whether authorities acted on account of the actual needs of the citizens. In Germany, several participants feared that at the level of policy-makers epidemiological concerns were taking primacy over economic ones, and that in the future, the economic consequences of the pandemic might outweigh the health-related consequences.

## Discussion

In this article, we investigated the types of uncertainties that the COVID-19 pandemic generated among members of the public and people with barriers to information, and how these uncertainties might have influenced responses to RCCE efforts. Four types of uncertainties emerged as potentially impacting the perception of, and response to, RCCE efforts. Our analysis shows that the uncertainties experienced during the pandemic were complex, and that they permeated across socio-economic contexts and societal groups. While various types of uncertainties preoccupied key informants and communities during the COVID-19 pandemic, there was a clear divergence between what policy makers and individuals from affected communities considered to be relevant uncertainties that needed to be addressed during the outbreak.

### Communication of uncertainty

Stakeholders involved in RCCE attempted to address epidemiological uncertainties related to the nature of the virus. In other words, they focused on the uncertainties surrounding the science of the SARS-CoV-2 virus, which is consistent with attitudes described in previous viral outbreaks [48]. However, our analysis found that people still experienced epidemiological uncertainties regarding the transmission patterns of the virus and its severity. This, in turn, seemed to have impended on the comprehensibility of regulations implemented to contain the spread of the virus. Furthermore, particularly prevalent during focus group discussions were non-epidemiological uncertainties: information uncertainties, social uncertainties, and economic uncertainties. Our findings indicate that both epidemiological and non-epidemiological uncertainties can have a negative influence on RCCE efforts when unaddressed, because they influence people’s ability to assess the risk and can be detrimental in terms of the trust people place in containment efforts.

### Non-epidemiological uncertainties, trust, and risk assessment

Previous work shows that for mitigation efforts during a pandemic to be successful, risk communication needs to enable trust, which is crucial in legitimizing decisions made by authorities [49]. RCCE guidelines recommend acknowledging uncertainty to reduce uncertainty-induced stress and fear and enhance trust [50, 51]. In line with this recommendation, key informants from all four socio-economic contexts highlighted that acknowledging uncertainty was an important attribute of regional and national RCCE strategies during COVID-19. However, our analysis also suggests that many of the uncertainties people experienced, and that were relevant in terms of their reaction to RCCE, remained unaddressed, risking a decrease in compliance and trust in containment efforts among affected communities. Our findings show that in Nigeria and Guinea, unaddressed uncertainties correlated with people’s trust in the authorities’ response to the pandemic. Our analysis also shows that in the two high-income countries (Singapore and Germany), unaddressed uncertainties did not seem to correlate as much with the public trust in the pandemic response. Future research could help to understand the factors which mediate the relationship between unaddressed uncertainties and public trust during pandemics.

Furthermore, one of the key aims of risk communication is to ensure public engagement in mitigation efforts by enabling people to assess risk and make informed decisions to protect themselves and their loved ones [51]. Risk perception is an important determinant of an individual’s protective behavior ([52], cited in [53]), which means that the inability to accurately assess risks can lead to disengagement in pandemic mitigation efforts. In fact, our analysis indicates that experienced uncertainties might have hampered people’s ability to accurately assess the risks posed by the pandemic and to act accordingly. Failure to address and resolve uncertainties might thus have contributed to limited participation in the pandemic response.

### Barriers to individual and communal efforts to manage uncertainties

In addition, research focusing on information seeking behaviors during the Zika pandemic found that attempts to resolve uncertainties included turning to authoritative sources of medical information (such as family doctors, national ministries of health and national public health institutions, and international organizations), but also to information from friends and family [54, 55]. Focus group participants from all four countries showed similar information-seeking behaviors, yet our analysis suggests that such efforts to resolve uncertainties are hampered by two interrelated types of uncertainties: First, information-seeking was complicated by what we call information uncertainty, notably by the high amount of available information – or what the WHO calls an “infodemic” [56]– and the dynamic changes of regulations and recommendations. Both factors made it difficult for individuals to identify reliable sources of information to deal with their uncertainties. Second, the search for reassurance through information-seeking was hampered by the lack of predictability of the pandemic and the lack of information about how to adjust to a changed social environment, unclear future prospects, and potential negative economic and societal impacts. In the case of vulnerable communities, the convergence of multiple uncertainties seemed to translate into a sense of existential threat, further increasing experienced uncertainties and aggravating the situation in terms of both trust and risk assessment. The examples of migrant communities in Germany and Singapore reiterate that during times of crisis, culturally sensitive approaches need to be deployed to inform vulnerable groups [57], beyond translating information from the official language to that of the targeted population.

### Implications for risk communication

Until high levels of natural or vaccine-induced immunity are reached, the best ways to contain the spread of the pandemic remain non-pharmaceutical interventions, which means that RCCE is key in containing COVID-19 [58]. Drawing on lessons learned during the H1N1 pandemic from 2009 to 2010 MacPhail argued that mitigation efforts can be strengthened if relevant authorities addressed uncertainties related to the spread and nature of the virus in their communication [59]. McPhail explained that an efficient way to retain scientific authority and manage uncertainty was presenting the unknowns of the virus as opportunities for learning and by gradually resolving such uncertainties. Our study provides insights for future communication strategies that would address uncertainties beyond those deemed relevant by scientific communities and local authorities and that are more aligned with the experiences of affected communities. The divergence between uncertainties experienced by research participants and those addressed in RCCE efforts could stand as proof of the effectiveness of addressing those uncertainties, which were the focus of RCCE campaigns. Hence, our recommendation is to broaden the scope of uncertainty-related messages in RCCE efforts during future public health emergency events. Our study draws attention to the importance of including non-epidemiological uncertainties, such as information-, social-, and economic uncertainties. In fact, our findings show that similar types of uncertainties take different shapes in different socio-economic contexts, and that adapting messages to fit individual contexts should be considered during future outbreaks. Furthermore, the uncertainties we describe are not exhaustive, and other types may emerge as relevant in future pandemic events.

### Strengths and limitations

The main strengths of our study represent the comparison of diverse sociocultural, economic, political, and epidemiological contexts as well as the large amount of data obtained through KII and FGDs. However, this study has a number of limitations. First, interview guides for both key informants and FGD did not primarily focus on uncertainties and their influence on RCCE efforts. The relevance of this topic emerged during the first line of analysis. The nature of this study is thus exploratory, indicating the importance of considering and further investigating uncertainties during a pandemic. Second, there is no consensus about which uncertainties would have to be considered during a public health emergency [13] that could have guided us in our analysis. To circumvent these difficulties, we used a grounded theory approach to establish the framework for analysis. Indeed, scientists from various disciplines have proposed frameworks for analyzing uncertainty [17]. The framework we advance overlaps somewhat with previous models from Han and colleagues [17, 22] and notably with that of Afifi and Afifi [36, 47]. However, we believe the framework we propose complements existing models in several important ways. For instance, the types of uncertainties we recommend emerged from a comparative analysis of four very different cultural, political, and socio-economic contexts. Further, we developed the framework to specifically refer to COVID-19, around uncertainty and risk communication. Also, previously developed frameworks aimed to introduce recommendations for uncertainty management at the individual and community level [36, 60]. Our study complements these suggestions by drawing attention to relevant messages that could be addressed by stakeholders and communicators during health emergency events. We consider the established framework one of the strengths of our study. Third, our analysis focused on how experienced uncertainties influenced local and national RCCE efforts from the perspective of recipients and might, therefore, be subject to negativity bias. Previous anthropological studies of epidemics suggest that experienced uncertainties can also have positive effects, such as the preservation of hope during a crisis [61, 62]. Positive effects were not apparent in our analysis and should be explored in future studies. Fourth, the divergence between responses of focus group participants with barriers to information and those from support organizations working with them might be indicative of a “halo” effect – of an attempt to give answers presumed to be expected – in participants’ responses. Future inter-disciplinary studies could combine FGD with ethnographic methods of data collection to determine which answers are given because they were presumed to be “correct” and how they differ from the actual experiences of people, as well as adding information from before and after FGDs.

## Conclusion

Uncertainty is a defining characteristic of the COVID-19 pandemic [10]. To our knowledge, this is one of the first comparative studies that explores uncertainties experienced by members of the public as well as by those responsible for RCCE during the COVID-19 outbreak in four different socio-economic contexts. Our findings suggest that both epidemiological and non-epidemiological uncertainties, when unaddressed by RCCE, can influence risk assessment, trust in, and compliance with public health measures. In future outbreaks, communicators and policy makers could improve the risk assessment and the trust of affected communities by acknowledging, addressing, and gradually trying to resolve both epidemiological and non-epidemiological uncertainties.

## Data Availability

Due to the qualitative nature of the data, the confidentiality agreements signed, and the ease with which respondents might be identified based on the content of the transcripts, we are unable to make the interview transcripts publicly available. A copy of the consent form can be requested from the corresponding author.
